# Alkaline water improves exercise-induced metabolic acidosis and enhances anaerobic exercise performance in combat sport athletes

**DOI:** 10.1371/journal.pone.0205708

**Published:** 2018-11-19

**Authors:** Jakub Chycki, Anna Kurylas, Adam Maszczyk, Artur Golas, Adam Zajac

**Affiliations:** 1 Department of Sports Training, the Jerzy Kukuczka Academy of Physical Education in Katowice, Katowice, Poland; 2 Department of Methodology and Statistics, The Jerzy Kukuczka Academy of Physical Education in Katowice, Katowice, Poland; University of Kentucky Medical Center, UNITED STATES

## Abstract

Hydration is one of the most significant issues for combat sports as athletes often use water restriction for quick weight loss before competition. It appears that alkaline water can be an effective alternative to sodium bicarbonate in preventing the effects of exercise-induced metabolic acidosis. Therefore, the main aim of the present study was to investigate, in a double blind, placebo controlled randomized study, the impact of mineral-based highly alkaline water on acid-base balance, hydration status, and anaerobic capacity. Sixteen well trained combat sport athletes (n = 16), were randomly divided into two groups; the experimental group (EG; n = 8), which ingested highly alkaline water for three weeks, and the control group (CG; n = 8), which received regular table water. Anaerobic performance was evaluated by two double 30 s Wingate tests for lower and upper limbs, respectively, with a passive rest interval of 3 minutes between the bouts of exercise. Fingertip capillary blood samples for the assessment of lactate concentration were drawn at rest and during the 3^rd^ min of recovery. In addition, acid-base equilibrium and electrolyte status were evaluated. Urine samples were evaluated for specific gravity and pH. The results indicate that drinking alkalized water enhances hydration, improves acid-base balance and anaerobic exercise performance.

## Introduction

Despite numerous scientific data, there is still no conclusive answer regarding what and how much we should drink to optimize sports performance. Until the middle of the 20^th^ century, the recommendation was to avoid drinking to optimize performance. The first drinking guidelines were introduced by the ACSM to avoid heat stress in 1975, while hydration and performance were first addressed only in 1996 [1]. At that time, athletes were encouraged to drink the maximum amount of fluids during exercise that could be tolerated without gastrointestinal discomfort and up to the rate lost through sweating. Depending on the type of exercise and the environment, volumes from 0.6 to 1.2 L per hour were recommended. These drinking guidelines have been questioned recently, and other issues such as over hydration and hyponatremia have been addressed [2].

The inconsistency of the results regarding hydration and sports performance arise from differences in experimental protocols. In studies in which dehydration develops during exercise, fluid loss of up to 4% body mass does not compromise performance, while in studies that induced dehydration prior to exercise, performance impairments have been observed after dehydration as low as 1–2% body mass [3]. Several comprehensive reviews on the influence of dehydration on muscle endurance, strength, anaerobic capacity, jumping performance and skill performance in team sport games have revealed negative effects of dehydration ≥ 2% body mass [4, 5, 6]. Hydration is one of the most significant issues for combat sports, as athletes often use water restriction for quick weight loss before competition. During tournaments lasting several hours, combat sport athletes sweat immensely and increase their core temperature affecting muscle strength, reducing motor cortex activation, peripheral stimulus as well as the speed of reaction and power output [7].

Considering the vast amounts of fluids used during exercise, water seems to be the most often form of hydration. Water comes in different forms, with specific properties depending on its mineral content. The pH of water, as well as the proportions between SO_4_^2-^ and HCO_3_^-^ determines hydration status and other therapeutic properties [7]. Drinking hydrogen rich water in human nutrition is a rather new concept, and it is recently suggested for medical purposes and hydration during exercise [8–10]. Alkaline water is being marketed as a nutritional aid for the general public for acidity-lowering, antioxidant, and antiaging properties. Some of the animal and human research has confirmed its effectiveness as an alkalizing agent in the treatment of metabolic acidosis [11, 12]. However, metabolic acidosis that occurs during high intensity exercise is a distinct form of metabolic alteration, when cells are forced to rely on anaerobic ATP turnover that leads to proton release and a decrease in blood pH that can impair performance [8, 13].

Anaerobic exercise metabolism leads to the production of lactic acid in the working muscles. Part of the produced lactic acid is released to the blood, reducing blood pH, and disturbing acid—base balance. Several studies have provided evidence that hydrogen ions are released from the muscles in excess of lactate after intense exercise [14]. Two mechanisms have been proposed to explain this phenomenon. It seems that hydrogen ions are released both by a sodium-hydrogen ion exchanger and by a lactic acid transporter [15]. Since red blood cells have a higher buffering capacity than blood plasma, the lactate generated during exercise largely remains in the plasma while hydrogen ions are transferred to the red blood cells and buffered by hemoglobin [16]. One of the objectives of training and supplementation in high intensity anaerobic sports disciplines is to increase the buffering capacity of the blood and tissues [17]. The use of sodium bicarbonate has proven effective in speed endurance and strength endurance sports, yet its use has been limited due to the possibility of gastrointestinal distress, metabolic alkalosis, and even edema due to sodium overload [8, 18]. It appears that alkaline water can be an effective alternative to sodium bicarbonate in preventing exercise-induced metabolic acidosis [8, 19]. Contrary to bicarbonate, alkaline water can be used on an everyday basis and has no known side effects. However, there are only few cross-sectional or longitudinal studies on the impact of alkaline water ingestion in combat sport athletes. Therefore, the main objective of the current study was to investigate in a double blind, placebo controlled randomized study, the impact of mineral-based highly alkaline water on acid-base balance, hydration status, and anaerobic capacity in experienced combat sport athletes subjected to a very intense exercise protocol.

## Materials and methods

### Subjects

Sixteen very well-trained males, who trained and competed in combat sports for at least 7.6 years, participated in the study. The athletes constituted a homogenous group in regard to age (average age of 22.3 ± 0.5 years), somatic characteristics, as well as aerobic and anaerobic performance (Table 1). The subjects (n = 16) were randomly divided into two groups, the experimental group (EG; n = 8), which received highly alkaline water, and the control group (CG; n = 8), which was hydrated with table water. All subjects had valid medical examinations and showed no contraindications to participate in the study. The athletes were informed verbally and in writing of the experimental protocol, the possibility to withdraw at any stage of the experiment, and gave their written consent for participation. The study was approved by the Research Ethics Committee of the Academy of Physical Education in Katowice, Poland.

**Table 1:** Characteristics of the study participants.

Variables	Experimental Group (n = 8)	Control Group (n = 8)
Age (yrs.)	22.7±3.2	22.4 ± 2.8
Height (cm)	181.2±2.1	178.3±4.9
Body mass (kg)	81.8±3.2	79.2 ±2.6
FM (%)	10.2±2.1	10.8±2.4
W_t—upper limbs_ (J/kg)	138±14	136±19
W_t—lower limbs_ (J/kg)	276±04	283±26
P_max–lower limbs_ (W/kg) P_max–upper limbs_ (W/kg)	19.8±0.9 8.9±1.1	20.2±1.6 8.7±0.4
VO_2max_ (ml/kg/min)	64.7±2.8	62.6±3.2

### Diet and hydration protocol

Energy intake, as well as macro and micronutrient an intake of all subjects was determined by the 24 h nutrition recall 3 weeks before the study was initiated. The participants were placed on an isocaloric (3455 ± 436 kcal/d) mixed diet (55% carbohydrates, 20% protein, 25% fat) prior and during the investigation. The pre-trial meals were standardized for energy intake (600 kcal) and consisted of carbohydrate (70%), fat (20%) and protein (10%). During the experiment, and 3 weeks before the commencement of the study, the participants did not take any medications or supplements. Throughout the experiment water intake was individualized based on the recommendation of the National Athletic Trainers Association and averaged 2.6–3.2 L per day. In our study we used water which had a pH of 9.13 which is highly alkaline compared to other commercially available products. The water ingested during the experiment contained 840 mg/dm^3^ of permanent ingredients, and was classified as medium mineral content. The bicarbonate ion HCO_3_^-^ (357.8 mg/dm^3^) and carbonate ion CO_3_^2-^ (163.5 mg/dm^3^) consisted the dominant anions. Sodium (Na^+^ 254.55 mg/dm^3^) dominated among cations. The water contained bicarbonate, carbonate-sodium (HCO_3_^-^, CO_3_^-^ Na^+^). The chemical properties of both types of water used in the experiment (alkaline and table water) are presented in Table 2.

**Table 2:** Chemical properties of water used in the study.

Variable	Measurement Unit	Alkaline Water	Table Water
pH	pH	9.13 ± 0.04	5.00 ± 0.08
CO_3_^2-^	mg/dm^3^	163.5 ± 6.3	14.98 ± 0.66
HCO_3_^-^	mg/dm^3^	357.8 ± 6.14	3.62 ± 0.12
Cl^-^	mg/dm^3^	26.4 ± 2.3	0.41 ± 0.03
SO_4_^2-^	mg/dm^3^	7.81± 1.2	1.60 ± 0.09
Na^+^	mg/dm^3^	254.55 ± 7.1	1.21 ± 0.05
K^+^	mg/dm^3^	0.91 ± 0.04	0.30 ± 0.03
Ca^2+^	mg/dm^3^	10.00 ± 1.6	1.21 ± 0.05
Mg^2+^	mg/dm^3^	0.37 ± 0.03	0.40 ± 0.04

Note: Data shows mean values ± SD of three analysis of each type of water

### Study protocol

The experiment lasted 3 weeks, during which two series of laboratory analyses were performed. The tests were carried out at baseline and after three weeks of hydration with alkaline or table water. The study was conducted during the preparatory period of the annual training cycle, when a high volume of work dominated the daily training loads. The participants refrained from exercise for 2 days before testing to minimize the effect of fatigue.

The subjects underwent medical examinations and somatic measurements. Body composition was evaluated in the morning, between 8.00 and 8.30 am. The day before, the participants had the last meal at 20.00. They reported to the laboratory after an overnight fast, refraining from exercise for 48h. The measurements of body mass were performed on a medical scale with a precision of 0.1 Kg. Body composition was evaluated using the electrical impedance technique (Inbody 720, Biospace Co., Japan). Anaerobic performance was evaluated by a two double 30-second Wingate test protocol for lower and upper limbs respectively, with a passive rest interval of 3 minutes between the bouts of exercise. The test was preceded by a 5 min warm-up with a resistance of 100 W and cadence within 70–80 rpm for lower limbs and 40 W and 50–60 rpm for the upper limbs. Following the warm-up, the test trial started, in which the objective was to reach the highest cadence in the shortest possible time, and to maintain it throughout the test. The lower limb Wingate protocol was performed on an Excalibur Sport ergocycle with a resistance of 0.8 Nm·Kg-1 (Lode BV, Groningen, Netherland). The upper body Wingate test was carried out on a rotator with a flying start with a load of 0.45 Nm·Kg-1 (Brachumera Sport, Lode, Netherland). Each subject completed 4 test trials with incomplete rest intervals. The variables of peak power–P_max_ (W/Kg) and total work performed–W_t_ (J/Kg), were registered and calculated by the Lode Ergometer Manager (LEM, software package, Netherland).

### Biochemical assays

To determine lactate concentration (LA), acid-base equilibrium and electrolyte status the following variables were evaluated: LA (mmol/L), blood pH, pCO_2_ (mmHg), pO_2_ (mmHg), HCO_3- act_ (mmol/L), HCO_3-std_, (mmol/L), BE (mmol/L), O_2SAT_ (mmol/L), ctCO_2_ (mmol/L), Na^+^ (mmol/L), and K^+^ (mmol/L). The measurements were performed on fingertip capillary blood samples at rest and after 3 minutes of recovery. Determination of LA was based on an enzymatic method (Biosen C-line Clinic, EKF-diagnostic GmbH, Barleben, Germany). The remaining variables were measured using a Blood Gas Analyzer GEM 3500 (GEM Premier 3500, Germany).

Urine samples were taken at rest, after an overnight fast, at baseline and at the conclusion of the investigation. They were placed in a plastic container and mixed with 5 ml/L of 5% solution of isopropyl alcohol and thymol for preservation. Urine samples were assayed for the presence of blood and proteins. Specific gravity was determined using the Atago Digital refractometer (Atago Digital, USA). Urine pH was determined based on the standardized Mettler Toledo potentiometer (Mettler Toledo, Germany).

### Statistical analysis

The Shapiro-Wilk, Levene and Mauchly´s tests were used to verify the normality, homogeneity and sphericity of the sample’s data variances, respectively. Verifications of the differences between analyzed variables before and after water supplementation and between the EG and CG were performed using ANOVA with repeated measures. Effect sizes (Cohen’s d) were reported where appropriate. Parametric effect sizes were defined as large for d > 0.8, as moderate between 0.8 and 0.5, and as small for < 0.5 (Cohen 1988; Maszczyk et al., 2014, 2016). Statistical significance was set at p<0.05. All statistical analyses were performed using Statistica 9.1 and Microsoft Office, and were presented as means with standard deviations.

## Results

All participants completed the described testing protocol. All procedures were carried out in identical environmental conditions with an air temperature of 19.2°C and humidity of 58% (Carl Roth hydrometer, Germany).

The repeated measures ANOVA between the experimental and control group and between the baseline and post-intervention period (3 weeks of alkaline and table water ingestion) revealed statistically significant differences for thirteen variables (Table 3).

**Table 3:** Statistically significant differences between the experimental and control groups at baseline and after 3 weeks of intervention (alkaline vs table water).

Variables	d	p	F
Wingate Lower Limbs Average Power Exp.	0.884	0.001	21.161
Wingate Upper Limbs Average Power Exp.	0.587	0.011	8.528
Wingate UL Peak Power Exp.	0.501	0.026	6.228
Wingate LL Total Work Exp.	0.567	0.045	4.822
Wingate UL Total Work Exp.	0.522	0.011	8.459
LA _rest_	0.534	0.008	9.429
LA _post exr_	0.618	0.003	13.382
pH _rest_	0.834	0.001	120.159
HCO_3_^-^_rest_	0.844	0.001	109.250
HCO_3_^-^_post exr_	0.632	0.002	14.724
K^+^_post exr_	0.501	0.040	5.154
Urine pH	0.589	0.017	7.298
SG	0.884	0.001	19.707

**N**ote: d—effect size; p—statistical significance

F–value of analysis of variance function

**Fig 1:**
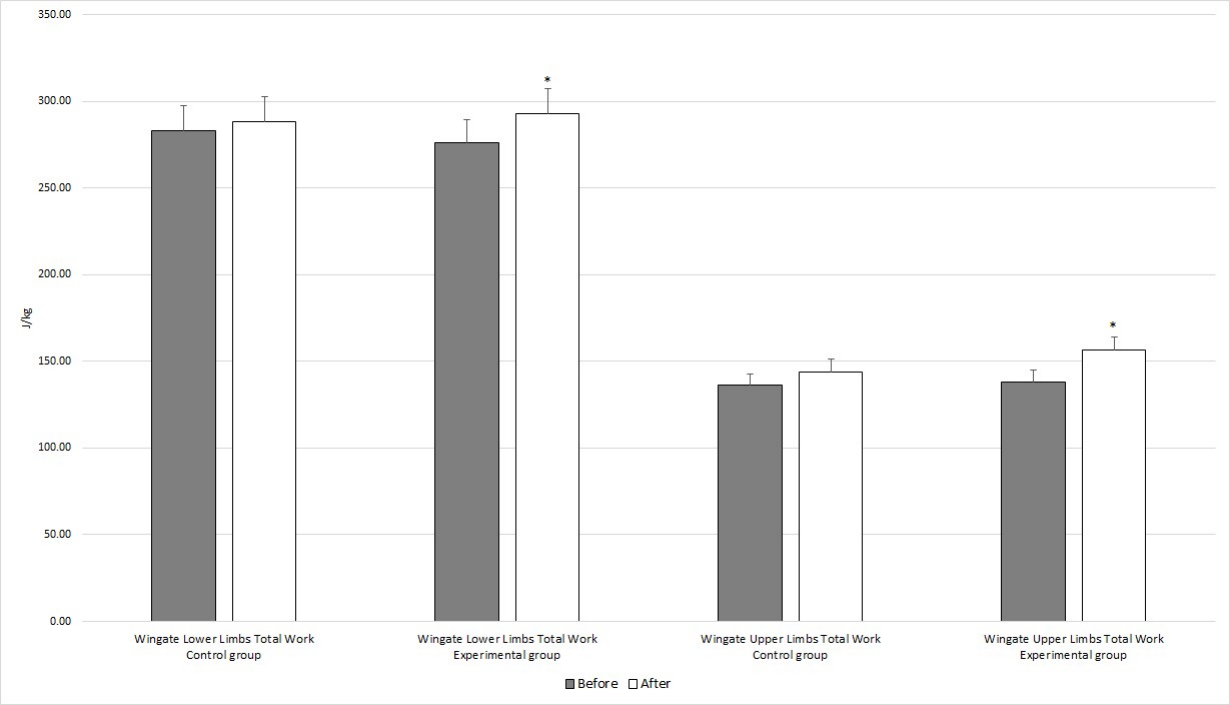
Differences between the control and experimental groups in total work of the lower and upper limbs (30s Wingate test) at baseline and after 3 weeks of alkaline or table water ingestion. Note: * statistically significant values.

Post-hoc tests revealed a statistically significant increase in mean power when comparing the values (7.98 J/kg to 9.38 J/kg with p = 0.001) at baseline vs. at the conclusion of the study in the experimental group supplemented with alkaline water. In contrast, the control group which received table water did not reveal any statistically significant results. Similar changes were observed for Upper Limb Average Power (from 4.32 J/kg to 5.11 J/kg with p = 0.011) and Upper Limb Peak Power (from 7.90 J/kg to 8.91 J/kg with p = 0.025) in the experimental group. The post-hoc tests also showed statistically significant increases in values for Lower Limb Total Work (from 276.04 J/kg to 292.96 J/kg with p = 0.012) and Upper Limb Total Work (from 138.15 J/kg to 156.37 J/kg with p = 0.012) when baseline and post intervention values were compared. The changes in the control group were not statistically significant. These results are presented in Fig 1.

Post-hoc tests also revealed statistically significant decreases in LA concentration at rest (from 1.99 mmol/L to 1.30 mmol/L with p = 0.008), and a significant increase in post exercise LA concentration (from 19.09 mmol/L to 21.20 mmol/L with p = 0.003) in the experimental group ingesting alkaline water.

Additionally, a significant increase in blood pH at rest (from 7.36 to 7.44 with p = 0.001), HCO_3_^-^ at rest (from 23.87 to 26.76 with p = 0.001), and HCO_3_^-^ post exercise (from 12.90 to 13.88 with p = 0.002) were observed in the experimental group. The other significant changes occurred in the post exercise concentration of K^+^ (from 4.15 to 4.41 with p = 0.039), in urine pH (from 5.75 to 6.62 with p = 0.017), and a decrease in the value of SG (from 1.02 to 1.00 with p = 0.001), all in the experimental group supplemented with alkaline water.

## Discussion

Acid-base equilibrium within the human body is tightly maintained through the blood and tissue buffering systems, the diffusion of carbon dioxide from the blood to the lungs via respiration, and the excretion of hydrogen ions from the blood to urine by the kidneys. These mechanisms also regulate acid-base balance following high intensity exercise. Metabolic acidosis is a consequence of exercise induced ionic changes in contracting muscles. Increased intramuscular acidity impairs muscle contractibility, significantly limiting high intensity exercise performance [20]. Importantly, acid-base equilibrium can be influenced by dietary supplementation.

In the present study, we investigated the effect of mineral-based alkaline water on acid-base balance, hydration status and anaerobic performance of competitive combat sport athletes. The study participants were experienced athletes (Table 1), capable of performing extreme anaerobic efforts. We have chosen such an approach for two reasons. First, it is well-documented that consumption of alkalizing water can have a significant effect on the hydration status, acid-base balance, urine and blood pH [8, 10], as well as Ca metabolism and bone resorption markers [21]. However, the majority of these research reports have been performed on sedentary individuals [22] or on subjects with self-reported physical activity [10]. Second, alkalization by alkaline water has been mostly discussed in the context of dehydration and aerobic performance [10]. Therefore, our study is novel by including both well trained combat sport athletes and the use of an extremely intensive anaerobic exercise protocol.

### Acid-base balance and hydration status

The exchange of ions, CO_2_, and water between the intracellular and extracellular compartments helps to restore acid-base balance following intensive exercise. There is sufficient data indicating that, supplements that modify the blood buffering system affect high-intensity exercise performance [23]. In humans, especially well trained athletes muscle pH may decrease from 7.0 at rest to values as low as 6.4–6.5 during exercise [24]. Ergogenic aids that help buffer protons attenuate changes in pH and enhance the muscle’s buffering capacity. This in turn allows for a greater amount of lactate to accumulate in the muscle during exercise.

The results of our study are in line with the available literature regarding the impact of alkaline water on blood and urine pH at rest [9, 19, 25]. However, novel results of the present research are related to the changes in HCO_3-_ after exercise in athletes ingesting alkaline water. Bicarbonate-CO_2_ accounts for more than 90% of the plasma buffering capacity. Supplementation can increase bicarbonate concentration in the blood and its pH. Since bicarbonate concentration is much lower in the muscles (10 mmol/L) than in the blood (25 mmol/L), the low permeability of charged bicarbonate ions precludes any immediate effects on muscle acid-base status [24]. These results confirm the view that an appropriate hydration status is necessary for active bicarbonate ion transport.

Several lines of evidence support the negative impact of dehydration (>2% body mass) on muscle endurance, strength, and anaerobic performance [6]. On the other hand, literature data indicates that consumption of alkaline water following a dehydrating bout of cycling exercise was shown to rehydrate cyclists faster and more completely compared to table water. Following consumption of alkaline water, the cyclists demonstrated lower total urine output, their urine was more concentrated (i.e., with higher specific gravity), and the total blood protein concentration was lower, indicating improved hydration status [26]. Our previous study revealed that the use of water with alkalizing properties exhibits a significant potential for hydration during anaerobic exercise [9]. The results of the present study confirm a decrease in urine specific gravity (from 1.02 to 1.00, with p = 0.001) and an increase in urine pH as the result of consumption of alkaline water. These results illustrate that the habitual consumption of highly alkaline water can markedly improve hydration status.

### Anaerobic performance

The current investigation demonstrated a significant increase in anaerobic capacity (W_t_−J/Kg) of athletes in the experimental group supplemented with alkaline water. The improvements in W_t_ following alkaline water consumption were influenced by positive changes in blood pH and bicarbonate. This phenomenon could be explained by the ergogenic effects of high alkalization and mineral ingredients.

High intensity exercise in which anaerobic glycolysis provides ATP for muscle contraction leads to an equal production of lactate and hydrogen ions. Most of the released hydrogen ions are buffered; however, a small portion (~0.001%) that remains in the cytosol causes a decrease in muscle pH and an impairment of exercise. Lactate efflux [15] and its oxidation are accompanied by a similar removal of hydrogen ions. The results of the current study demonstrated a statistically significant decrease in lactate concentration at rest (from 1.99 mmol/L to 1.30 mmol/L, p = 0.008), and a significant increase post exercise (from 19.09 mmol/L to 21.20 mmol/L, p = 0.003) when compared to the baseline levels with the values recorded at the end of alkaline water supplementation. The extremely intense 4 x 30s upper/lower limb Wingate test protocol employed in our study, with only short rest intervals between each bout of exercise, was a likely reason that less of the total lactate produced in the muscles was transported to the blood [27].

Muscle blood flow determines lactate efflux from the muscle [28], and is dependent on the activity of lactate transport proteins [29], the extracellular buffering capacity [30], and the extracellular lactate concentration [28]. Thus, our results on lactate concentration are in agreement with the view that anaerobic performance (i.e., W_t_−J/Kg, W_Avr_−J/Kg) depends on counter-regulatory variables. Indeed, we demonstrated that changes in resting blood pH and HCO_3_^-^ significantly improved anaerobic performance. Another variable that can affect anaerobic performance includes blood viscosity. Weidmann et al. (2016) showed that the intake of highly alkaline water decreased blood viscosity by 6.30%, compared to table water (3.36%) in 100 recreationally active female and male subjects. Therefore, it may be possible that the excess of metabolic end-products (namely, H^+^ and Pi), which disturb cellular homeostasis and muscle contraction, are more effectively transported. The available literature data does not specify clearly which components of buffering capacity are altered by the above changes. It must be indicated, that there are several methods available to determine muscle buffering capacity. Due to the methodological complexity, none of these methods are free from criticism. In most studies buffering capacity has been determined in vitro by titration, which does not include trans-membrane transport of acid-base substances or dynamic buffering by biochemical processes occurring in vivo [31].

Most studies show a documented ergogenic effect of bicarbonate loading during exhaustive exercise lasting 1–7 min, when anaerobic glycolysis plays a major role in energy provision [32]. The rationale for the ergogenic effect of bicarbonate is that the increase in extracellular pH and bicarbonate will enhance the efflux of lactate and H^+^ from muscle. There is also evidence that the ergogenic effect of bicarbonate is more pronounced during repeated sprints than during sustained exercise [30].

Different strategies used for improving buffering capacity of tissues and blood do not allow for a direct comparison. Despite this, there appears to exist an ergogenic effect in response to NaHCO_3_^-^, what may explain the large effect size noted by Tobias et al. [33]. In our research we obtained large effect sizes with regards to 4 variables (Average power of the lower limbs, resting HCO_3_^-^, resting blood pH and urine SG).

## Conclusions

The results of the present study indicate that drinking alkalized water improves hydration status, acid-base balance, and high intensity anaerobic exercise performance. It appears that both greater muscle buffering capacity and enhanced removal of protons, resulting in increased glycolytic ATP production, may be responsible for these effects. Considering the energy demands and the intense sweat rate of combat sport athletes, the authors recommend the daily intake of 3–4 L of highly alkaline mineralized water to improve hydration and anaerobic performance during training and competition.

## Supporting information

S1 Table Data for Fig 1. (XLSX)

S2 Table Stress test data. (XLSX)

S3 Table Water data. (XLSX)
